# Lanthanide-doped heterostructured nanocomposites toward advanced optical anti-counterfeiting and information storage

**DOI:** 10.1038/s41377-022-00813-9

**Published:** 2022-05-20

**Authors:** Yao Xie, Yapai Song, Guotao Sun, Pengfei Hu, Artur Bednarkiewicz, Lining Sun

**Affiliations:** 1 Department of Physics, College of Sciences, Shanghai University, Shanghai, 200444 China; 2 Department of Chemistry, College of Sciences, Shanghai University, Shanghai, 200444 China; 3 School of Materials Science and Engineering, Shanghai University, Shanghai, 200444 China; 4 Research Center of Nano Science and Technology, College of Sciences, Shanghai University, Shanghai, 200444 China; 5 Instrumental Analysis & Research Center, Shanghai University, Shanghai, 200444 China; 6 Institute of Low Temperature and Structure Research, Polish Academy of Sciences, 50-422 Wrocław, Poland

**Keywords:** Nanoparticles, Metamaterials

## Abstract

The continuously growing importance of information storage, transmission, and authentication impose many new demands and challenges for modern nano-photonic materials and information storage technologies, both in security and storage capacity. Recently, luminescent lanthanide-doped nanomaterials have drawn much attention in this field because of their photostability, multimodal/multicolor/narrowband emissions, and long luminescence lifetime. Here, we report a multimodal nanocomposite composed of lanthanide-doped upconverting nanoparticle and EuSe semiconductor, which was constructed by utilizing a cation exchange strategy. The nanocomposite can emit blue and white light under 365 and 394 nm excitation, respectively. Meanwhile, the nanocomposites show different colors under 980 nm laser excitation when the content of Tb^3+^ ions is changed in the upconversion nanoparticles. Moreover, the time-gating technology is used to filter the upconversion emission of a long lifetime from Tb^3+^ or Eu^3+^, and the possibilities for modulating the emission color of the nanocomposites are further expanded. Based on the advantage of multiple tunable luminescence, the nanocomposites are designed as optical modules to load optical information. This work enables multi-dimensional storage of information and provides new insights into the design and fabrication of next-generation storage materials.

## Abstract (web summary)

Lanthanide-doped heterostructured nanocomposites were developed by using cation exchange method and successfully applied toward advanced optical anti-counterfeiting and information storage

## Graphical abstract

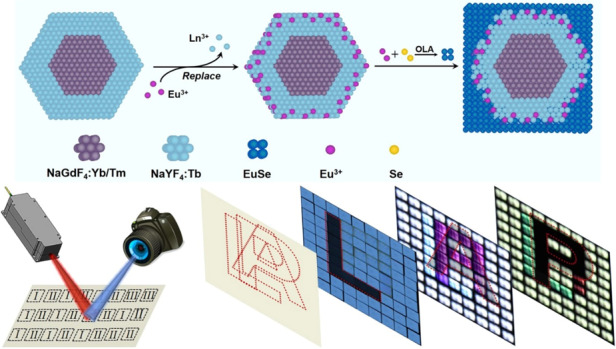

## Introduction

Currently, human society has entered the information age, in which our daily lives are being inundated by a huge amount of data. Recently years, researchers have focused on exploring innovative information storage devices and information storage strategies. Compared to the traditional magnetic storage and semiconductor memory, optical information storage exhibits the advantages of higher efficiency, lower energy consumption, longer storage life, and larger capacity, and thus it is widely considered as an important storage strategy for the next generation^1,2^.

To date, several kinds of luminescent materials, including metallic nanocrystals^3,4^, semiconductor quantum dots^5–8^, metal-organic frameworks^9–13^, organic dye molecules^14,15^, and lanthanide-doped nanomaterials^16–23^ have been developed as optical information storage media. Among them, lanthanide-doped nanomaterials have been considered as potential candidates because of their unique advantages of color-tunable luminescence^24,25^, long luminescence lifetime^26,27^, narrowband emission, and outstanding photochemical stability^28–30^. Due to the inherent 4f^n^ orbital and rich energy-level structures of lanthanide element, the lanthanide-doped nanomaterials exhibit excellent luminescent performance and efficient spectral conversion ability through down-shifting and upconverting processes^31^. They can emit polychromatic radiation covering the entire visible and near-infrared (NIR) spectral region when irradiated by NIR, visible, or ultraviolet (UV) light. However, the traditional single-model luminescent materials used for optical information storage have shortcomings such as small information storage capacity, which drives the urgent need to develop advanced luminescent materials as information carriers.

By integrating the stokes and anti-stokes processes into the same information carriers, the multimodal emission with adjustable colors could be achieved under the different excitation wavelength, which can increase the information storage capacity of luminescent materials. Therefore, the multi-mode luminescent materials are very promising in the field of information storage because of the characteristics of information storage capacity and security. In the previous research of dual-mode luminescent materials, a variety of lanthanide elements with upconversion and down-shifting luminescent properties are often doped in the same matrix^32–34^. This strategy caused the cross-relaxation between the lanthanides, which resulted in the decrease of luminescent intensity. We would like to combine Stokes and anti-Stokes features into one system. For the former, the semiconductors are efficient. For the latter, there are no better candidate than lanthanide-doped upconversion nanoparticles. However, the epitaxial growth is difficult between semiconductors and upconversion nanoparticles. Thus, it is promising to develop innovative heterostructured multi-mode luminescent materials with intentionally designed color or well-defined tunable properties for optical information storage.

**Scheme 1:**
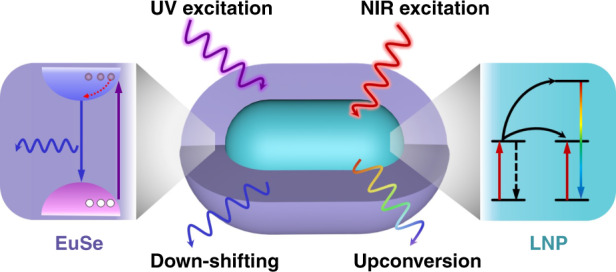
Schematic illustration of the down-shifting (UV excitation) and upconversion (NIR excitation) luminescence principle of heterostructured nanocomposite composed of lanthanide-doped upconversion nanoparticle (LNP) and EuSe semiconductor

Herein, we develop a heterostructured, colloidal nanocomposite made of EuSe semiconductor and lanthanide-doped upconversion nanoparticle by utilizing a cation exchange strategy, which possess outstanding down-shifting and upconversion performances. Benefit from cation exchange strategy, the transition layer containing Eu^3+^ ions forms between the EuSe semiconductor and the lanthanide-doped upconversion nanoparticle, and the characteristic emission of Eu^3+^ ions can be detected under both UV and NIR light irradiation. As presented in Scheme 1, the nanocomposite could emit blue and white light under excitation of UV light, and also shows color modulation upconverting emission under excitation of 980 nm laser *via* controlling the type and content of lanthanide elements in the upconversion nanoparticles. In addition, the upconverting emission with a long lifetime from specific lanthanide ions could be filtered through time-gating technology. Stemming from the multimode luminescent properties of nanocomposites, we demonstrate their suitability for advanced anti-counterfeiting and information storage successfully.

## Results and discussions

**Fig. 1:**
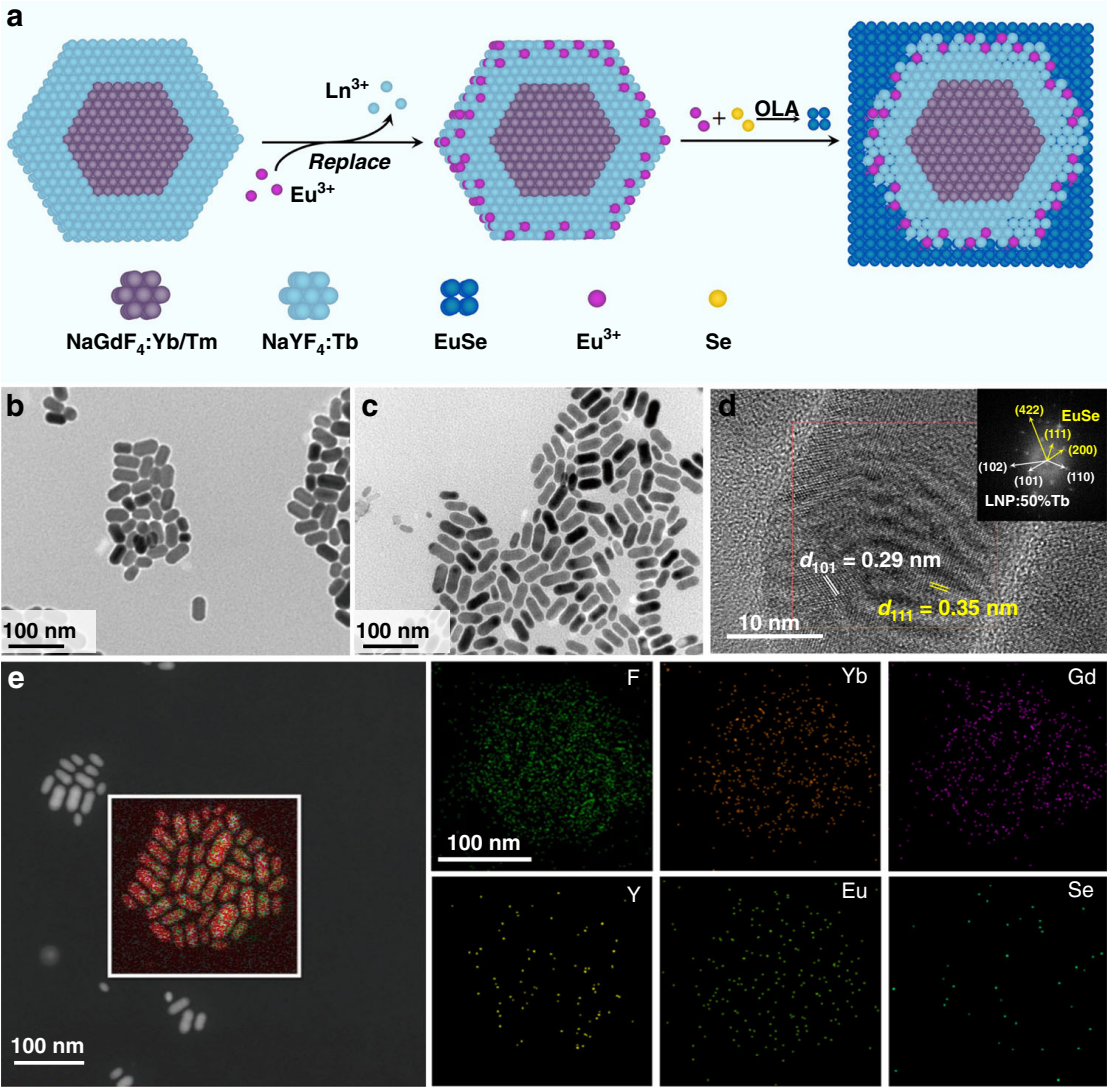
Synthesis of NaGdF_4_:Yb,Tm@NaYF_4_:50%Tb@EuSe (denoted as LNP:50%Tb@EuSe) nanocomposite. **a** Schematic diagram of the design of EuSe growing on NaGdF_4_:Yb,Tm@NaYF_4_:50%Tb (denoted as LNP:50%Tb). **b** TEM image of LNP:50%Tb. **c** TEM image of LNP:50%Tb@EuSe. **d** HRTEM image of LNP:50%Tb@EuSe. Inset shows the corresponding Fourier transform diffraction pattern. **e** Scanning transmission electron microscopy image and element mappings of F, Gd, Yb, Y, Eu, and Se elements for LNP:50%Tb@EuSe

We developed a methodology for combining EuSe semiconductors with upconversion nanoparticles by ions exchange between different lanthanide ions. Namely, the Tb^3+^ doped NaYF_4_ shell was homogeneously deposited on the NaGdF_4_:49%Yb,1%Tm upconverting core (denoted as NaGdF_4_:Yb,Tm), and in the critical step of our method the partial lanthanide ions (Tb^3+^, Y^3+^) on the surface of such NaGdF_4_:Yb,Tm@NaYF_4_:50%Tb nanoparticles were replaced by Eu^3+^ through cation exchange in the dispersion (Fig. 1a). After that, the oleylamine would be used as a reducing agent during the synthesis of EuSe, and the Eu^3+^ ions on the surface were partially reduced to Eu^2+^ ions which form the EuSe together with selenium powder^35–37^. It is generally considered that epitaxial growth is difficult when the lattice mismatch is large between two materials. In this case, the cation exchange of Eu^3+^ ions and other lanthanides could promote the formation of buffer layers to reduce the lattice mismatch and promote the heterogeneous epitaxial growth of EuSe^38,39^. As demonstrated by transition electron microscopy (TEM) images, the obtained NaGdF_4_:Yb,Tm cores show uniform spherical morphology (Fig. S1). The lanthanide-doped upconversion nanoparticle NaGdF_4_:Yb,Tm@NaYF_4_:50%Tb (denoted as LNP:50%Tb) and NaGdF_4_:Yb,Tm@NaYF_4_:50%Tb@EuSe (LNP:50%Tb@EuSe) nanocomposite display uniform rod-like morphology and monodispersed particle (Fig. 1b, 1c). The size distributions of LNP:50%Tb and LNP:50%Tb@EuSe were measured from different directions. As shown in Fig. S2, the length of LNP:50%Tb@EuSe nanocomposite (around 44.7 nm) is significantly longer than that of LNP:50%Tb (around 35.8 nm) after growth of EuSe. Meanwhile, the X-ray diffraction patterns of the LNP:50%Tb and LNP:50%Tb@EuSe nanocomposite, as illustrated in Fig. S3, show that the intensity of diffraction peaks attributed to LNP:50%Tb is significantly lower for the nanocomposite and a broad diffraction peak appears around 25 degree. The nanocomposite exhibits low crystallinity because of the formation of buffer layers during the epitaxial growth of EuSe^39,40^. The high-resolution TEM (HRTEM) image of LNP:50%Tb@EuSe nanoparticle was shown in Fig. 1d. The lattice fringes could be well resolved and a *d*-spacing of 0.29 nm can be observed, which is attributed to the (101) plane of hexagonal NaYF_4_. An additional *d*-spacing of 0.35 nm is attributed to the (111) plane of cubic EuSe. The inset of Fig. 1d reveals that the LNP:50%Tb@EuSe is a polycrystalline nanoparticle compounded by hexagonal phase NaYF_4_ and cubic phase EuSe.

It was reported that the lattice mismatch may lead to anisotropic shell growth and shape distortion^41,42^. In order to prove that the growth of EuSe on the surface of lanthanide-doped nanoparticles is anisotropic, we synthesized the spherical nanoparticles (NaYF_4_:20%Yb,0.5%Tm) with homogenous morphology as a matrix, and then EuSe grew on the surface. The changes in the morphology of nanoparticles during the growth of EuSe are investigated by TEM measurement of the samples collected after heat treatment at 290 °C for different duration (0 h, 1 h, 2 h, and 3 h, respectively). As shown in Fig. S4, the morphology of nanoparticles gradually transforms from spherical to rod-like with the increase of heat treatment time. This indicates that the growth of cubic EuSe on the surface of hexagonal upconversion nanoparticles is anisotropic.

The growth of EuSe leads to significant changes of ligands on the nanoparticle surface (Fig. S5). Only oleic acid groups are present on the surface of LNP:50%Tb, while oleic acid and oleylamine groups coexist on the surface of LNP:50%Tb@EuSe due to the successful growth of EuSe. At the same time, compared to LNP:50%Tb, the LNP:50%Tb@EuSe nanocomposite exhibits stronger absorption in the UV region (Fig. S6). These results further demonstrate the successful growth of EuSe on the surface of LNP:50%Tb. In addition, the energy dispersive X-ray spectroscopy (EDS) and element mappings of the nanocomposites verify the distribution of F, Yb, Gd, Y, Eu, and Se over the randomly selected region (Fig. 1e and Fig. S7). It can be observed that the content of Eu element in the nanocomposite is obviously more than that of Se element. This is due to the presence of partially unreduced trivalent europium ions in the LNP:50%Tb (see Fig. 1). The X-ray photoelectron spectroscopy (XPS) spectrum of the nanocomposite as well as high-resolution XPS spectrum at Eu 3d position of nanocomposite are shown in Fig. S8. The signals of Eu^2+^ 3*d*_5/2_, Eu^3+^ 3*d*_5/2_, Eu^2+^ 3*d*_3/2_ and Eu^3+^ 3*d*_3/2_ can be observed clearly, which illustrates that both Eu^2+^ and Eu^3+^ ions are coexistent in these nanocomposites.

**Fig. 2:**
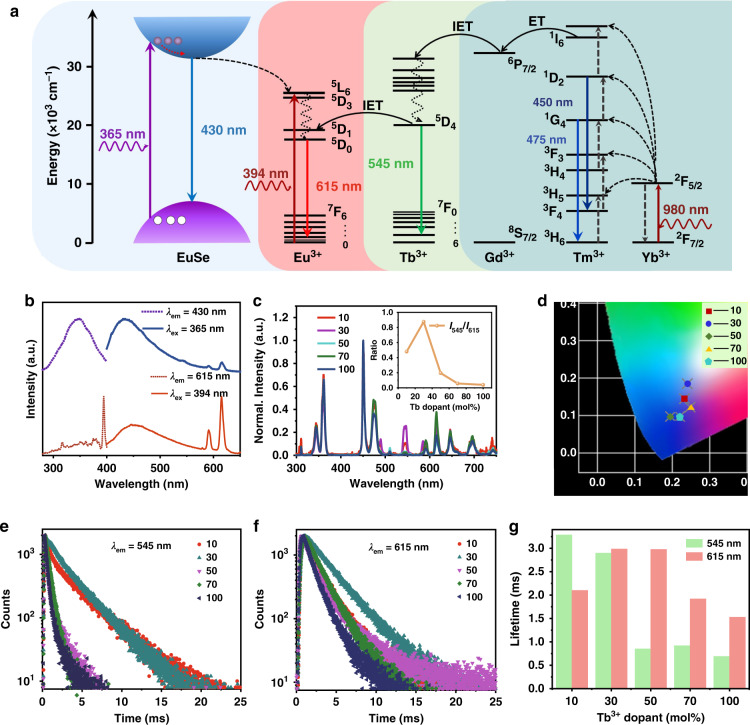
Optical investigations of NaGdF_4_:Yb,Tm@NaYF_4_:x%Tb@EuSe (denoted as LNP:x%Tb@EuSe) nanocomposites. **a** The energy transfer mechanism of LNP:x%Tb@EuSe nanocomposites for down-shifting luminescence under UV irradiation (365 and 394 nm, respectively) and upconversion emission under 980 nm laser irradiation. **b** Normalized excitation (blue dotted line, λ_em_ = 430 nm; and red dotted line, λ_em_ = 615 nm) and emission (blue solid line, λ_ex_ = 365 nm; and red solid line, λ_ex_ = 394 nm) spectra of LNP:50%Tb@EuSe. **c** Normalized upconversion emission spectra of LNP:x%Tb@EuSe (x = 10, 30, 50, 70, and 100) under 980 nm laser excitation (10 W cm^-2^). Inset shows the ratio of emission intensity at 545 nm (^5^D_4_ → ^7^F_5_ electronic transition of Tb^3+^) and 615 nm (^5^D_0_ → ^7^F_2_ electronic transition of Eu^3+^) with different concentrations of Tb^3+^ ions. **d** The CIE chromaticity diagram of LNP:x%Tb@EuSe (x = 10, 30, 50, 70, and 100) under 980 nm laser irradiation. Concentration dependent luminescence decay curves of (**e**) Tb^3+^ at ^5^D_4_ state (monitored at 545 nm) and (**f**) Eu^3+^ at ^5^D_0_ state (monitored at 615 nm) in the LNP:x%Tb@EuSe (*x* = 10, 30, 50, 70, and 100) under 980 nm pulsed laser excitation. **g** The luminescence average lifetime values of Tb^3+ 5^D_4_ state at 540 nm (green bars) and Eu^3+ 5^D_0_ state at 615 nm (orange bars) based on Fig. 2e, f, respectively

Because of the successful growth of EuSe on the surface of LNP:50%Tb, the nanocomposite can display different colors under different excitation wavelengths of UV light (Fig. 2a and b). Figure 2a displays a model that illustrates the energy transfer processes of Eu^2+^ to Eu^3+^ and the characteristic f–f transition emission of Eu^3+^ in the nanocomposite. The emission of Eu^2+^ and Eu^3+^ will be competing under UV light irradiation when Eu^2+^ and Eu^3+^ coexist in the nanocomposites^43,44^. As presented in Fig. 2b, the excitation spectrum displays a broadband peak with a maximum at 340 nm by monitoring the emission at 430 nm, which comes from the 4*f*^7^ → 4*f*^6^5*d*^1^ electronic transition of Eu^2+^. However, a sharp excitation peak at 394 nm is observed by monitoring the characteristic emission of Eu^3+^ ion at 615 nm, which is derived from the ^7^F_0_ → ^5^L_6_ transition of Eu^3+^. As shown in the emission spectrum under excitation with 365 nm, a strong broadband emission peak at 430 nm from Eu^2+^ dominates over a weak emission peak at 615 nm from ^5^D_0_ → ^7^F_2_ of Eu^3+^ can be observed. Inversely, under 394 nm excitation, which is well suited for the f–f transition in Eu^3+^, the narrowband ^5^D_0_ → ^7^F_J_ Eu^3+^ emission dominate the Eu^2+^ broadband emission at 450 nm. The color coordinate values of LNP:50%Tb@EuSe nanocomposite under different wavelengths of UV excitation were plotted in the Commission Internationale de l’Eclairage (CIE) chromaticity diagram. As illustrated in Fig. S9, the color coordinate is in the blue region under 365 nm excitation, while it shifts to around white region under 394 nm excitation.

**Fig. 3:**
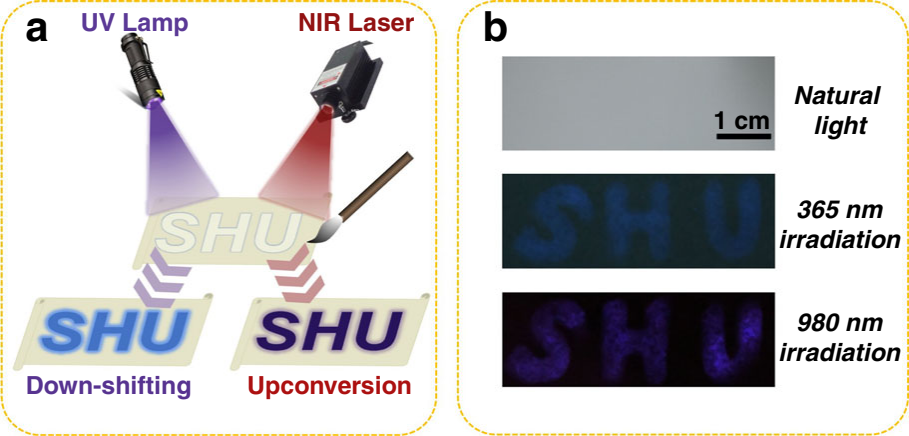
The experimental scheme of dual-mode anti-counterfeiting performance of LNP:50%Tb@EuSe. **a** “SHU” pattern drawn on the non-fluorescent paper by writing brush dipped in the fluorescent ink with LNP:50%Tb@EuSe. **b** Photographs of the “SHU” handwriting using fluorescent ink under the illumination of natural light, 365 nm UV lamp (80 mW cm^−2^) and 980 nm laser (500 mW cm^−2^), respectively

In addition, due to the energy transfer between Tb^3+^ and Eu^3+^ ions, we could change the type and content of Tb^3+^ ions in this system to control the luminescence of nanocomposites under 980 nm laser excitation^45,46^. In order to obtain multi-colored upconversion emission, we exploited the NaGdF_4_:Yb,Tm@NaYF_4_:x%Tb nanoparticles with different Tb content (LNP:x%Tb; *x* = 10, 30, 70, and 100, respectively) as a matrix to deposit EuSe aiming to design new multi-functional nanocomposites. The TEM images of these nanocomposites are shown in Fig. S10 and the down-shifting emission spectra in Fig. S11 (denoted as LNP:x%Tb@EuSe) demonstrate that the LNP:x%Tb are successfully coated by EuSe. Figure 2a also illustrates that an energy transfer pathway is successfully constructed by the introduction of Yb^3+^, Tm^3+^, Gd^3+^, Tb^3+^, and Eu^3+^ ions. Yb^3+^ ions are sensitizers that absorb the excitation light (980 nm) and transfer the energy to the high energy level of Tm^3+^ through energy transfer upconversion (ETU). The Gd^3+^ ions are used to receive energy from Tm^3+^ ions through the energy transfer (ET) approach and pass the energy to Tb^3+^ ions through interfacial energy transfer (IET). After that, part of the energy is released by radiative transitions (Tb^3+^ emission at 545 nm) and the other part is transferred to the Eu^3+^, leading to its characteristic emission at 615 nm. According to the power-dependent measurements (Fig. S12), the respective emissions of Gd^3+^, Tm^3+^, Tb^3+^, and Eu^3+^ at 311, 345, 545, and 615 nm are all governed by a five-photon process. Fig. S13a and Fig. 2c display the upconversion emission spectra of LNP:x%Tb and LNP:x%Tb@EuSe nanocomposites (*x* = 10, 30, 50, 70, and 100; λ_ex_ = 980 nm), respectively. By comparison, the characteristic emissions of Eu^3+^ ions are only observed in the spectra of LNP:x%Tb@EuSe, not in LNP:x%Tb. The 450 and 475 nm emission peaks are ascribed to the ^1^D_2_ → ^3^F_4_ and ^1^G_4_ → ^3^H_6_ electronic transition of Tm^3+^ ions, respectively. The 545 nm emission peak can be attributed to ^5^D_4_ → ^7^F_5_ electronic transition of Tb^3+^ ions and the 615 nm emission peak is derived from ^5^D_0_ → ^7^F_2_ electronic transition of Eu^3+^ ions. It can be observed that the emission intensities of both LNP:x%Tb and LNP:x%Tb@EuSe nanocomposites are changed obviously with different content of Tb^3+^ ions doped. Accordingly, the emission colors of LNP:x%Tb and LNP:x%Tb@EuSe display significant changes as well, owing to the difference of Tb^3+^ content (Fig. 2d and Fig. S13b). These results demonstrate that we achieve the color tuning by changing the relative contribution of Tm, Tb, and Eu upconversion emission in the nanocomposites by modulating the concentration of Tb^3+^ ions in the intermediate layer. The detailed investigation shows that the upconversion emission intensity of Tb^3+^ and Eu^3+^ is closely dependent on the concentration of Tb^3+^ in the interlayer which is in accordance with predictions. This is because under 980 nm excitation, the purposefully designed energy transfer sequence has been implemented, where Yb^3+^ → Tm^3+^ upconversion and Gd^3+^ population is followed by energy migration through Gd^3+^ network from the core to the shell dopants, which is then responsible for the population of Tb^3+^ excited states and ultimately feeds Eu^3+^ ions. The emission intensity ratio of Tb^3+^ (545 nm) to Eu^3+^ (615 nm) maximizes with Tb^3+^ at 30 mol% for the LNP:x%Tb@EuSe (Inset in Fig. 2c). It means that the rates of spontaneous emission and nonradiative energy migration vary with the content of Tb^3+^ ions^46^.

To further investigate the nonradiative energy migration and radiative emission in this system, the luminescence decay curves of Tb^3+^ at ^5^D_4_ state (monitored at 545 nm) (Fig. 2e) and Eu^3+^ at ^5^D_0_ state (monitored at 615 nm) (Fig. 2f) in LNP:x%Tb@EuSe (*x* = 10, 30, 50, 70, and 100) were measured under 980 nm pulse excitation. The results of the corresponding lifetime values were displayed and recorded in Fig. 2g and Table S1. The lifetime at ^5^D_4_ state of Tb^3+^ decreases rapidly with the increase of Tb^3+^ concentration, and the lifetime at ^5^D_0_ state of Eu^3+^ reaches a maximum at a doping content of 30 mol% of Tb^3+^. Thus, combined with the spectral results, the following energy transfer model can be deduced: the efficiency of nonradiative energy migration is low, and the energy is mainly used to emit through spontaneous emission for a lower Tb^3+^ content (<30 mol%); the high Tb^3+^ concentration results in a smaller dopant separation that increases the concentration quenching and promotes nonradiative energy migration^47–49^. Moreover, the lifetimes of upconversion emission from Tb^3+^ and Eu^3+^ (Fig. 2e, f) are much longer than that of Tm^3+^ when the content of Tb^3+^ is low (<30 mol%) (Fig. S14 and Table S1). Similarly, the luminescence lifetime of Tb^3+^ at ^5^D_4_ state (monitored at 545 nm) and Tm^3+^ at ^1^G_4_ state (monitored at 475 nm) shows the same changing feature in LNP:x%Tb (Fig. S15 and Table S2). In these nanoparticles, the raise of the concentration of Tb^3+^ ions also causes concentration quenching and thus promotes nonradiative relaxation. This characteristic makes it possible to store and decode the information by utilizing time-gating techniques (Fig. S16).

The upconversion luminescence quantum yields of lanthanide-doped nanoparticles are generally very low and less than 1.0 %^50,51^. For the LNP:50%Tb@EuSe nanocomposites, the down-shifting luminescence quantum yield was determined to be 5.226 % when dispersed in cyclohexane by taking rhodamine B as a reference^52,53^. Such quantum yield is sufficient to support the use of nanocomposites for optical anti-counterfeiting. To prove the feasibility of the heterostructured nanocomposite in the optical anti-counterfeiting application, the dual-modal luminescent images based on the upconversion and down-shifting multiplexing were designed. Without further processing, the LNP:50%Tb@EuSe nanocomposites dispersed in cyclohexane were used as the fluorescent ink. As depicted in Fig. 3a, we used a writing brush dipped in fluorescent ink to draw patterns on non-fluorescent paper. The pattern is invisible under natural light, but it is visible when illuminated by specific light sources. The UV light is from a portable UV lamp and the NIR light is from a 980 nm laser equipped with a beam expander. As presented in Fig. 3b, the “SHU” is not visible under natural light, while it emits blue and purple light when irradiated using UV (365 nm) and NIR light (980 nm, 500 mW cm^-2^), respectively. This demonstrates the potential of the nanocomposites for anti-counterfeiting applications.

**Fig. 4:**
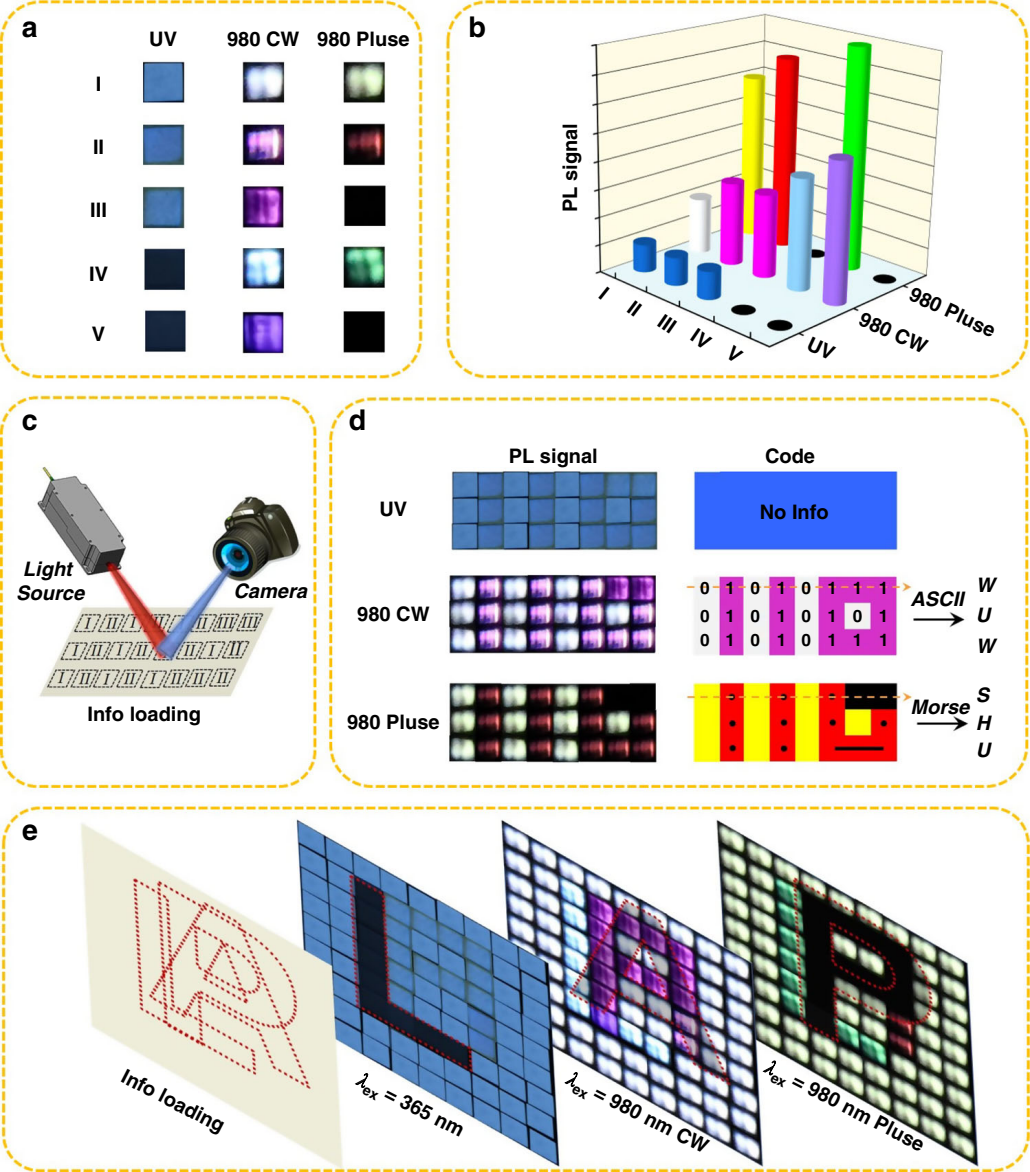
Loading and decoding of optical information. **a** Photos of different nanomaterials coated on a paper (1 × 1 mm) under illumination of UV Lamp (365 nm, 80 mW cm^-2^), 980 nm continuous wave (980 nm CW, 10 W cm^-2^) and 980 nm pulsed wave (980 nm Pulse), respectively. (I: LNP:30%Tb@EuSe; II: LNP:50%Tb@EuSe; III: LNP:100%Tb@EuSe; IV: LNP:50%Tb; V: LNP:100%Tb) **b** The corresponding three-dimensional histogram of the optical signals shown in **(a**). **c** Schematic diagram of optical information loading and decoding via establishing optical modulate array by using I, II, and III. **d** The photoluminescence (PL) signals of (**c**) under excitation of UV Lamp, 980 nm CW and 980 nm Pulse, respectively. The signals were converted into ASCII code and *Morse* code, and then pre-loading information of “WUW” and “SHU” would be read out, respectively (The orange dotted arrows mean the direction of information decoding). **e** This array of optical modules (9 × 9) containing the hidden information of “L”, “A”, and “P” could be decoded after irradiation of UV, 980 nm CW, and 980 nm Pulse, respectively

Encouraged by the good luminescent properties, we selected five different types of the lanthanide-doped nanoparticles and nanocomposites to investigate their suitability for applications in optical information storage. These five kinds of materials dispersed in cyclohexane were used to uniformly paint small (1×1 mm) square markings on non-fluorescent paper (Fig. 4a). They generate eight different visible photoluminescence signals under UV, 980 nm continuous wave (980 nm CW), and 980 nm pulsed laser (980 nm Pulse) excitation, respectively, which is also exhibited as the three-dimensional histogram for the optical signal (Fig. 4b). Every single piece of painted marking coated with luminescence material could be used as a single module with encoded optical information. The different information modules can be assembled in a specific order to store the information and collect the optical signals in different excitation modes. As illustrated in Fig. 4c, three types of optical information modules coated with LNP:x%Tb@EuSe (x = 30, 50, and 100) nanocomposites are manually arranged into a specific array (3 × 8). All the modules emit visible blue light when exposed to the UV light. However, the three types of modules emit different optical signals when the excitation modes were changed to 980 nm CW or 980 nm Pulse (Fig. 4d). On the one hand, the pre-stored information “WUW” could be output via converting the photoluminescence signal into 8-bit ASCII code (λ_ex_ = 980 nm CW). On the other hand, “SHU” could be read by converting the luminescence signal into *Morse* code (λ_ex_ = 980 nm Pulse). The characteristics of multi-dimensional information load greatly improve the security of information.

The modularity of luminescent materials can greatly increase the capacity of information storage. Not only can we convert photoluminescence signals into codes for advanced information encryption, we also can store the information that we would like to hide via an ordered arrangement of modules under specific excitation mode. As a proof of concept, we created three different modular matrices (9 × 9) and stored the “C”, “H”, and “N” into different matrices, respectively (Fig. S17). Moreover, we could also store three different messages simultaneously into one array. As shown in Fig. S18 and Fig. 4e, “L”, “A”, and “P” could be read respectively under the three excitation modes (UV, 980 nm CW, and 980 nm Pulse). At the macroscopic level, we are able to use the multi-mode luminescence of nanocomposites to store different information. In contrast to current popular laser printing multi-dimensional anti-counterfeiting and information storage technologies^54,55^, the advantage of the nanocomposites for information storage is that they are more flexible and require less quality of light source. If nanocomposites are loaded in an orderly manner on a substrate using single-particle printing technology, then a 1 × 1 mm substrate can be loaded with approximately 10^9^ nanocomposites, which would be a truly high-capacity optical information storage. Thus, these interesting results further demonstrate that the nanocomposites show excellent potential to store information.

## Conclusion

In summary, a series of heterostructured LNP:x%Tb@EuSe nanocomposites were synthesized, in which the cubic EuSe was grown anisotropically on the surface of hexagonal LNP:x%Tb. The nanocomposites emit blue and white down-shifting emission under 365 nm and 394 nm UV irradiation, respectively. This is due to the fact that the excitation efficiencies of Eu^2+^ and Eu^3+^ are different at different excitation wavelengths and the energy transfer between excited states of Eu^2+^ and Eu^3+^. Meanwhile, the nanocomposites also show tunable upconversion photoluminescence under NIR laser irradiation (λ_ex_ = 980 nm). In addition, the emission with a longer luminescence lifetime was filtered via the time-gating technique. Based on the whole feature, the nanocomposites were used for dual-mode anti-counterfeiting and optical information storage. More importantly, a larger information capacity could be achieved when nanomaterials are fabricated into information modules. The results confirm the great potential of the designed LNP:x%Tb@EuSe nanocomposites for advanced anti-counterfeiting and information storage applications. The simple but powerful route provides new perspectives for the development of advanced optical materials and the method of information storage.

## Materials and methods

### Chemicals and materials

YCl_3_·6H_2_O (99.99%), GdCl_3_·6H_2_O (99.99%), YbCl_3_·6H_2_O (99.99%), TmCl_3_·6H_2_O (99.99%), TbCl_3_·6H_2_O (99.99%), EuCl_3_·6H_2_O (99.9%), Selenium (Se, 99.99%), Tri-n-octylphosphine (TOP, 90%), Oleylamine (OLA, 90%) and methanol (CH_3_OH, 99.5%) were acquired from Shanghai Aladdin Biochemical Technology Co. Ltd. Oleic acid (OA, 90%), 1-octadecene (ODE, 90%) and Rhodamine B (analytical standard) were acquired from Sigma-Aldrich Co. Ltd. Ethanol (99.7%), cyclohexane (99.7%), ammonium fluoride (NH_4_F, 96%), and Sodium hydroxide (NaOH, 96%) were purchased from Sinopharm Chemical Reagent Co. Ltd. Deionized water is prepared in the lab and used during the whole experiment. All the chemicals were used without further purification.

### Synthesis of NaYF_4_:20%Yb,0.5%Tm core

NaYF_4_:20%Yb,0.5%Tm core nanoparticles were synthesized and collected according to the previous publication^8^.

### Synthesis of NaGdF_4_:Yb,Tm@NaYF_4_:x%Tb (denoted as LNP:x%Tb)

NaGdF_4_:Yb,Tm nanoparticles were synthesized based on the previous publication^46^. The system contained GdCl_3_·6H_2_O, YbCl_3_·6H_2_O, and TmCl_3_·6H_2_O at particular ratio (50:49:1 mol%). NaGdF_4_:Yb, Tm@NaYF_4_:x%Tb nanoparticles were synthesized by using the NaGdF_4_:Yb, Tm nanoparticles as seeds. In detail, ODE (15 mL) and OA (6 mL) were put into a 100 mL flask, then 1 mL water solution containing YCl_3_·6H_2_O and TbCl_3_·6H_2_O at designed ratios were added (Total amount is 0.5 mmol). The mixture was heated to 140 °C for 1 h and then cooled down to 50 °C under vacuum. Subsequently, 0.5 mmol NaGdF_4_:Yb, Tm nanoparticles along with 10 mL methanol solution containing NaOH (2 mmol) and NH_4_F (3 mmol) were added into the flask and stirred for 30 min. The solution was heated to 120 °C under vacuum for 40 minutes and then heated at 300 °C for 1.5 h under an argon flow. The NaGdF_4_:Yb,Tm@NaYF_4_:x%Tb nanoparticles were collected and washed as the same as the NaGdF_4_:Yb, Tm, and dispersed in cyclohexane (5 mL).

### Synthesis of LNP:x%Tb@EuSe

Firstly, 0.8 mmol of Se was added to the TOP (800 μL), and Se-TOP was synthesized by ultrasonic treatment for 20 minutes. Subsequently, EuCl_3_·6H_2_O (0.2 mmol), ODE (30 mL), OA (0.3 mL), OLA (3 mL) and pre-synthesized Se-TOP were put into three-necked flask (100 mL). The system was heated to 120 °C under an argon flow for 2.5 h. Then 0.4 mmol LNP:x%Tb were added in the above mixture and the temperate was raised to 290 °C for 3 h. The resulting LNP:x%Tb@EuSe nanocomposites were collected by centrifugation, rinsed with ethanol, and dispersed in 4 mL cyclohexane.

### Measurement of quantum yield

The downshifting luminescence quantum yield (φ) of LNP:50%Tb@EuSe nanocomposites dispersed in cyclohexane was determined relative to rhodamine B dissolved in methanol. The quantum yield was calculated using the following equation:

$${{{\mathrm{\varphi }}}} = {{{\mathrm{\varphi }}}}_{\rm{RB}} \times \frac{{I_{\rm{LNP}}}}{{I_{\rm{RB}}}} \times \frac{{E_{\rm{RB}}}}{{E_{\rm{LNP}}}} \times \frac{{\rm{Absorbance(RB)}}}{{\rm{Absorbance(LNP)}}} \times \frac{{n_{\rm{LNP}}^2}}{{n_{\rm{RB}}^2}}$$

where φ_*RB*_ is the luminescence quantum yield of rhodamine B, *I*_LNP_ and *I*_RB_ stand for integrated luminescence intensity of the LNP:50%Tb@EuSe nanocomposites and rhodamine B (RB), respectively. *E* is the excitation irradiance, *Absorbance* is the absorbance at the excitation wavelength (the nanocomposites were excited with 365 nm and rhodamine B was excited with 348 nm using the same power density), and n is the refractive index of the corresponding solvent.

### Characterization

The emission spectra and luminescence decay curves were recorded by an Edinburgh FS − 5 fluorescence spectrometer equipped with Xenon lamp and external 980 nm laser [MDL-III-980 nm, cnilaser (Changchun New Industries Optoelectronics Technology Co., Ltd, China)]. The X-ray diffraction (XRD) patterns were collected on 18 KW D/MAX2200 V PC X-ray powder diffractometer. Transmission electron microscopy (TEM) images were collected on JEM-200CX (200 kV). High-resolution transmission electron microscopy (HRTEM), energy-dispersive X-ray spectroscopy (EDS), Scanning transmission electron microscopy and element mapping images were collected on a JEM-2100F (200 kV). X-ray photoelectron spectroscopy (XPS) was measured with an ESCALAB 250Xi. Fourier transform infrared spectroscopy (FT-IR) spectra were recorded by an Avatar 370 in a range of 3500–500 cm^−1^ with KBr pressed tablets. UV–Vis absorption spectra were recorded by a Shimadzu UV-2600i ultraviolet-visible spectrometer in a range of 300–700 nm. The photos were taken by a digital camera (Canyon, EOS 5D Mark IV, ISO is 1000 and the acquisition time is 5 ms).

## Supplementary information

Supporting Information
