# Supplementary material for: Lanthanide-doped heterostructured nanocomposites toward advanced optical anti-counterfeiting and information storage
Source: Light Sci Appl. 2022 May 20;11:150. doi: 10.1038/s41377-022-00813-9 (PMC9122995; doi:10.1038/s41377-022-00813-9)
Supplement: Supplementary file 1 — Supporting Information [file 41377_2022_813_MOESM1_ESM.docx]

**Supplementary Information**

**for**

**Lanthanide-doped Heterostructured Nano****composites toward Advanced** **Optical** **Anti-Counterfeiting and Information Storage**

**Yao Xie^1,2^,** **Yapai Song^3,4^,** **Guotao Sun^3,4^,** **Pengfei Hu^5^,** **Artur Bednarkiewicz^6^ and Lining Sun^2,3,4^***

Correspondence: Lining Sun ([lnsun@shu.edu.cn](mailto:lnsun@shu.edu.cn))

^1^ Department of Physics, College of Sciences, Shanghai University, Shanghai 200444, China

^2^ Department of Chemistry, College of Sciences, Shanghai University, Shanghai 200444, China

^3^ School of Materials Science and Engineering, Shanghai University, Shanghai 200444, China

^4^ Research Center of Nano Science and Technology, College of Sciences, Shanghai University, Shanghai 200444, China

^5^ Instrumental Analysis & Research Center, Shanghai University, Shanghai 200444, China

^6^ Institute of Low Temperature and Structure Research, Polish Academy of Sciences, 50-422 Wrocław, Poland


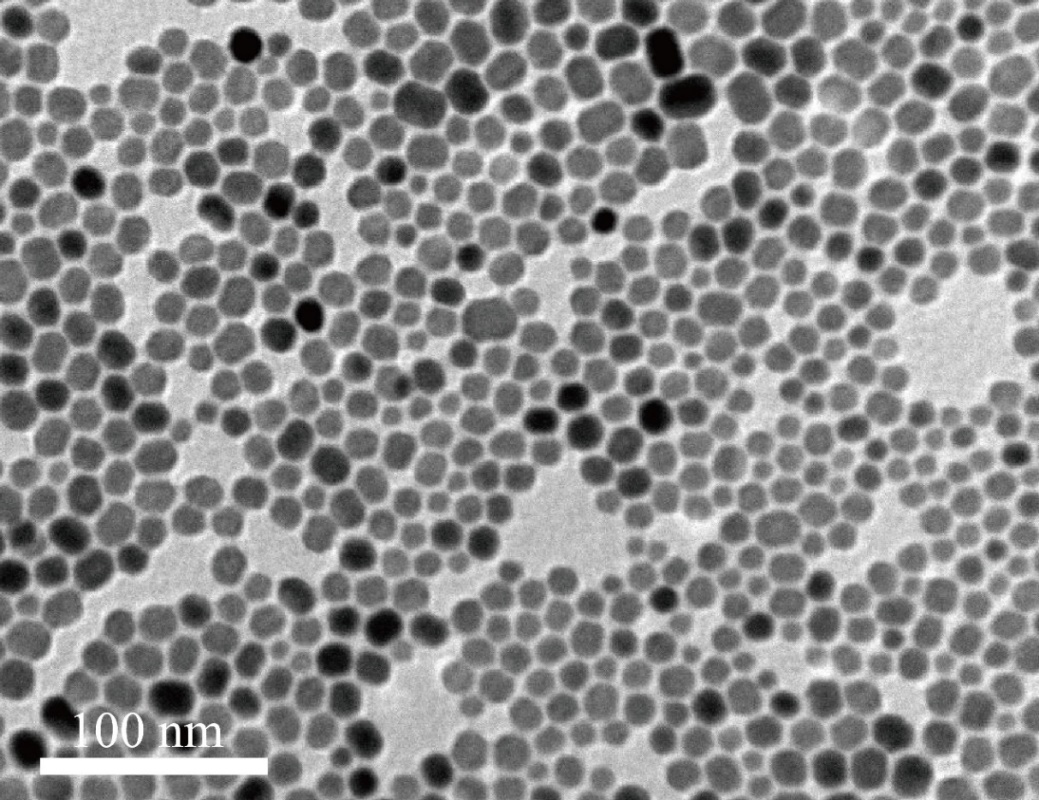


**Figure S1.** Transmission electron microscopy (TEM) image of NaGdF_4_:Yb,Tm nanoparticles.


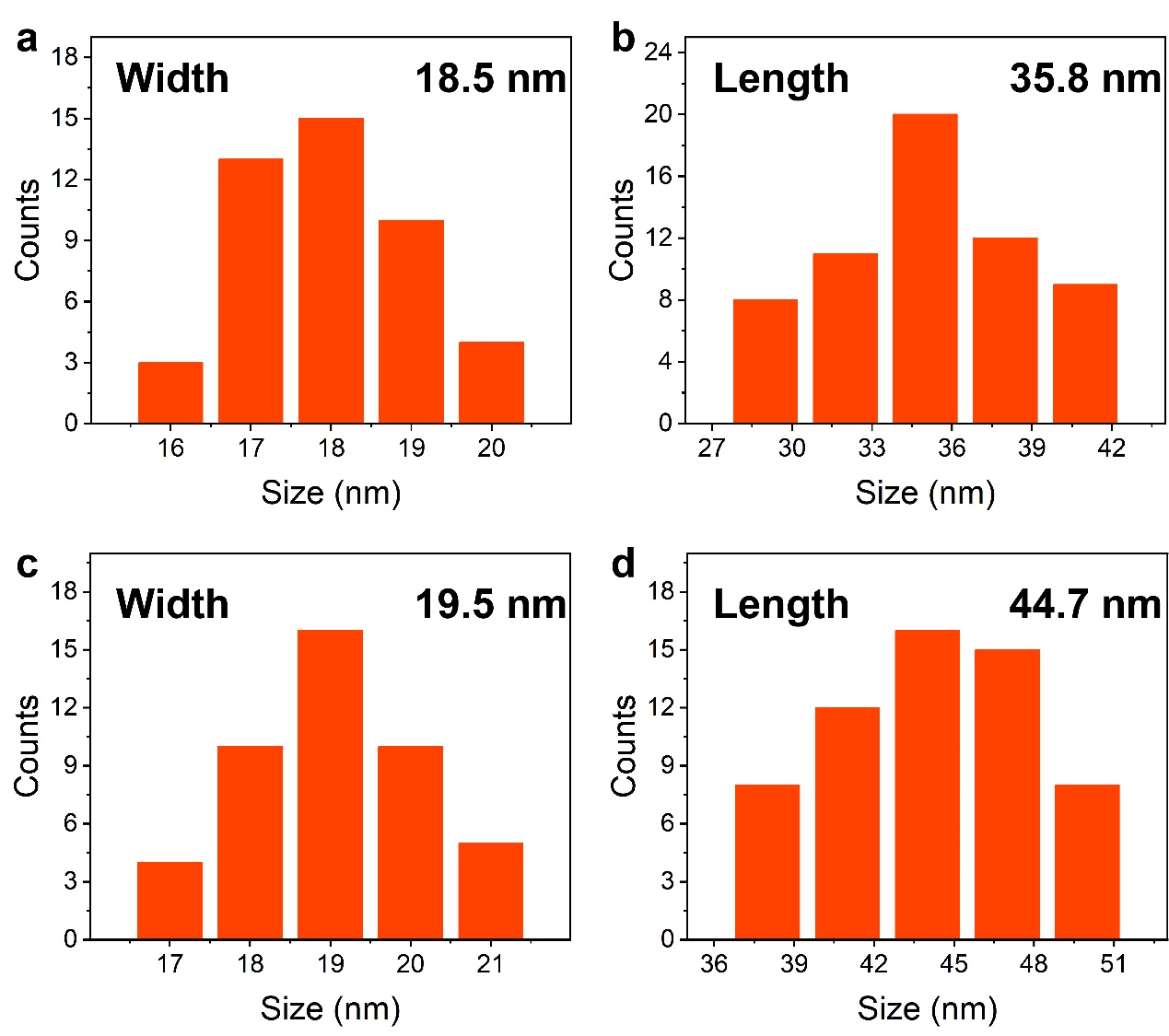


**Figure S2** Size distribution of LNP:50%Tb nanoparticles in different directions **(a) and (b).** The sizes were determined from the TEM image shown in Fig. 1b. Size distribution of LNP:50%Tb@EuSe nanocomposite in different directions **(c) and (d)**. The sizes were determined from the TEM image shown in Fig. 1c.


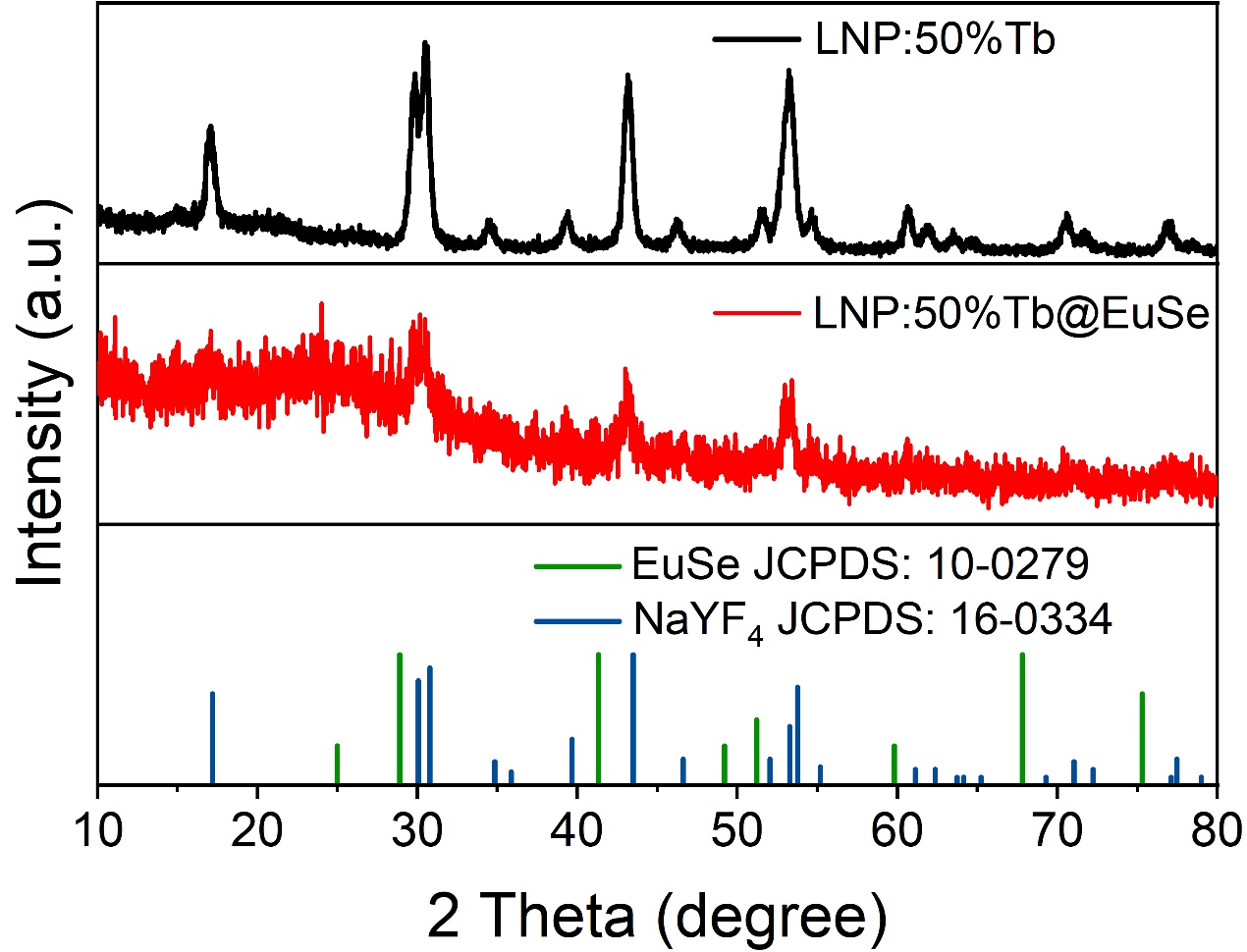


**Figure S3.** X-ray diffraction (XRD) patterns of NaGdF_4_:Yb,Tm@NaYF_4_:50%Tb (denoted as LNP:50%Tb) nanoparticles and LNP:50%Tb@EuSe nanocomposites. The standard JCPDS cards of hexagonal NaYF_4_ crystal and cubic EuSe crystal are given in the bottom.


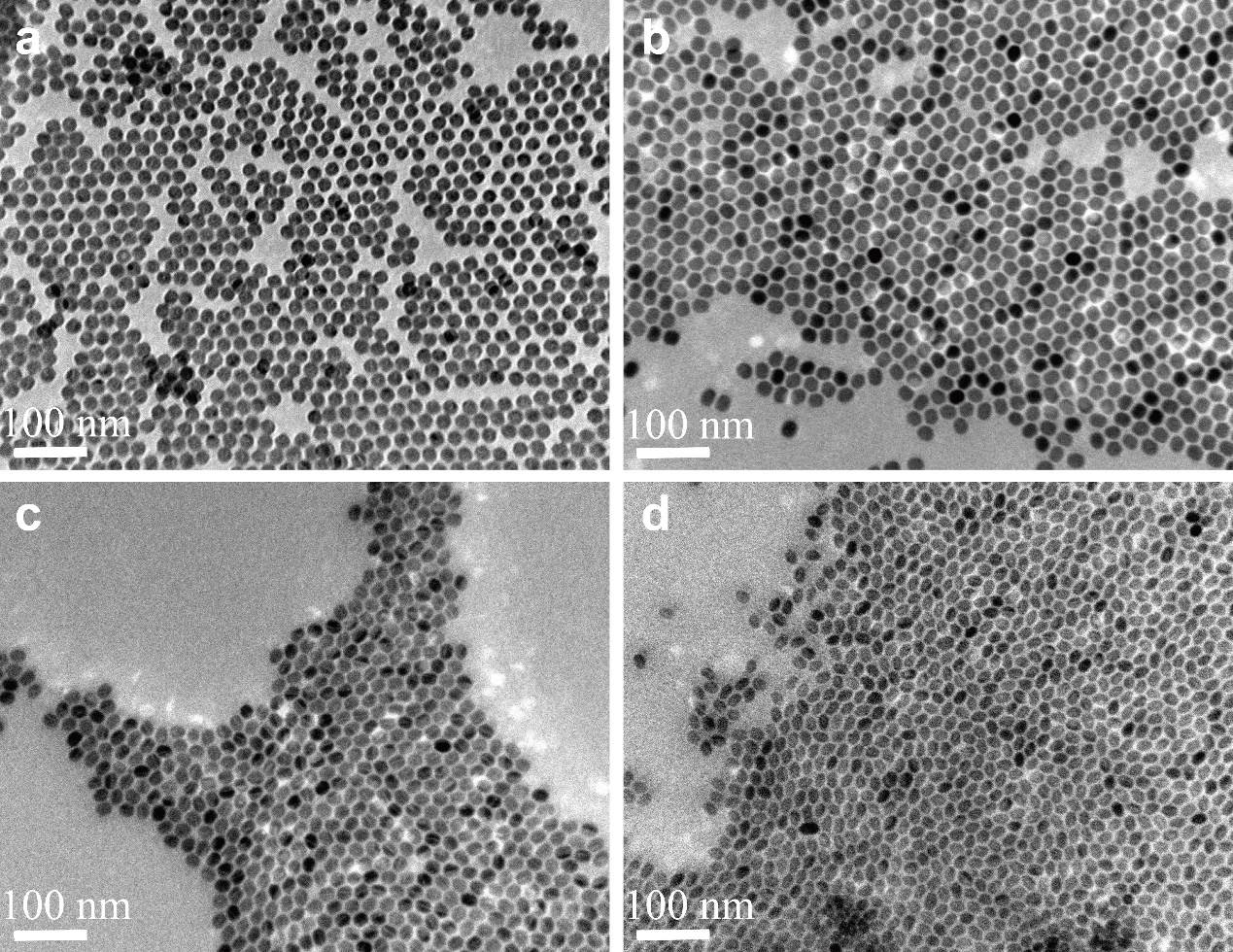


**Figure S4.** TEM images of NaYF_4_:20%Yb,0.5%Tm@EuSe nanocomposites collected during the synthesis after being heated at 290 °C for 0 h **(a),** 1 h **(b)**, 2 h **(c)**, and 3 h **(d)**.


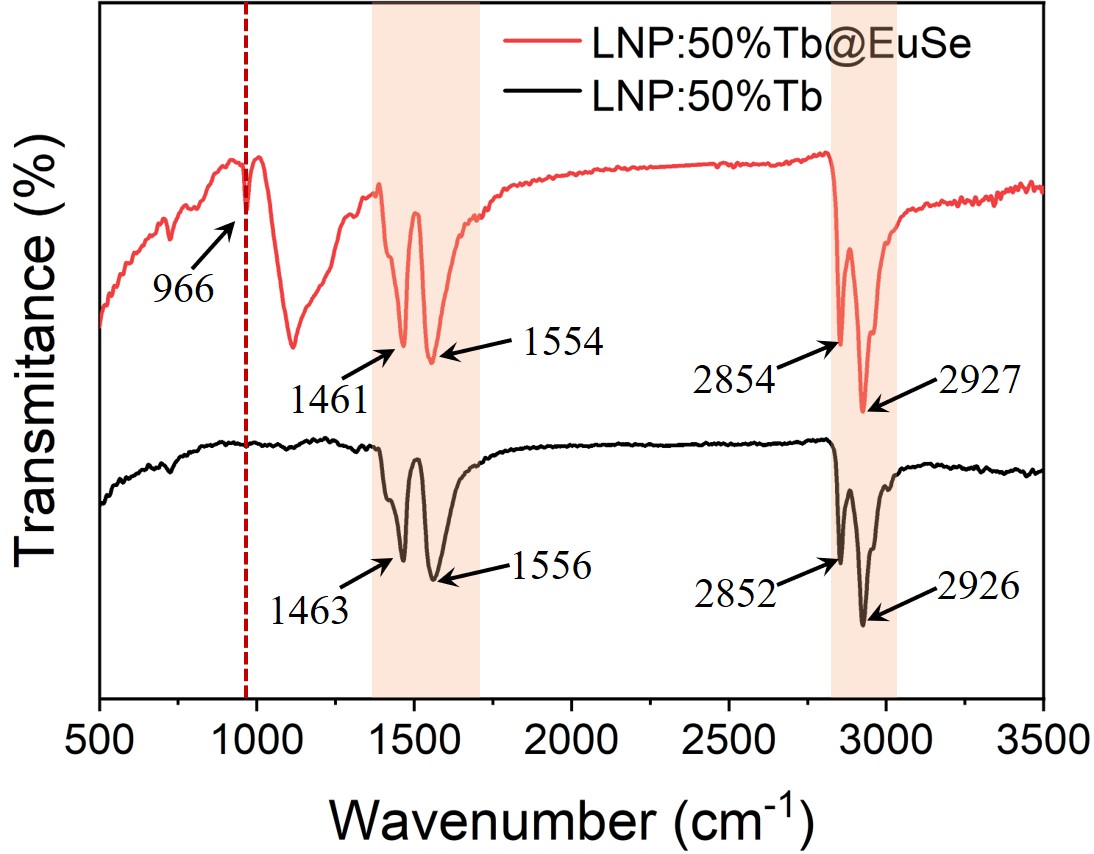


**Figure S5.** Fourier transform infrared (FTIR) spectra of LNP:50%Tb and LNP:50%Tb@EuSe nanocomposite. For the FTIR spectrum of LNP:50%Tb@EuSe, the peaks at 2854 cm^-1^ and 2927 cm^-1^ are assigned to acyclic C-H stretching. The peaks at 1554 cm^-1^ and 1461 cm^-1^ are attributed to C=O stretch and carboxylate (COO^-^) stretch. In the curve of LNP:50%Tb@EuSe, there is an additional characteristic peak at 966 cm^-1^ compared to that of LNP:50%Tb. This is indexed to C-N stretch of oleylamine. These results demonstrate that only oleic acid groups are present on the surface of LNP:50%Tb, while oleic acid and oleylamine groups coexist on the surface of nanocomposite.


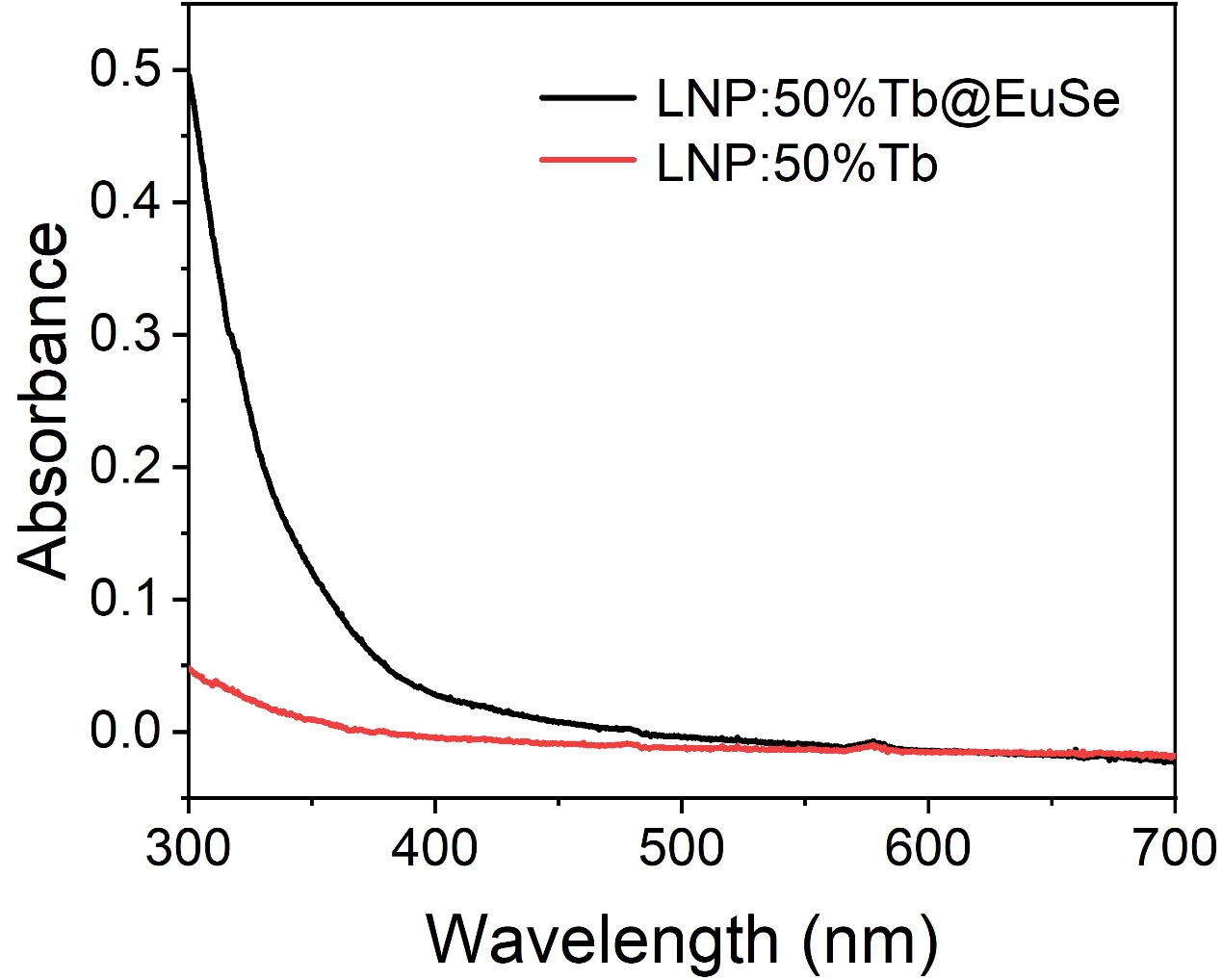


**Figure S6.** The absorption spectra of LNP:50%Tb and LNP:50%Tb@EuSe nanocomposite.


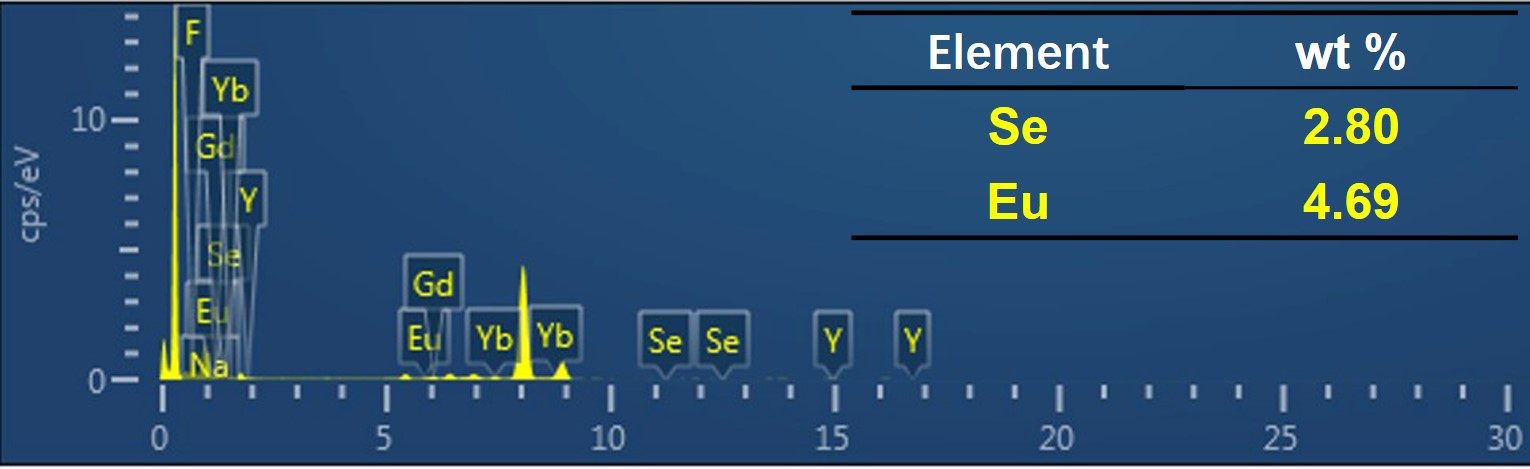


**Figure S7.** Energy dispersive X-ray spectroscopy (EDS) spectrum of LNP:50%Tb@EuSe nanocomposite. The content of Eu element is bigger than that of Se element.


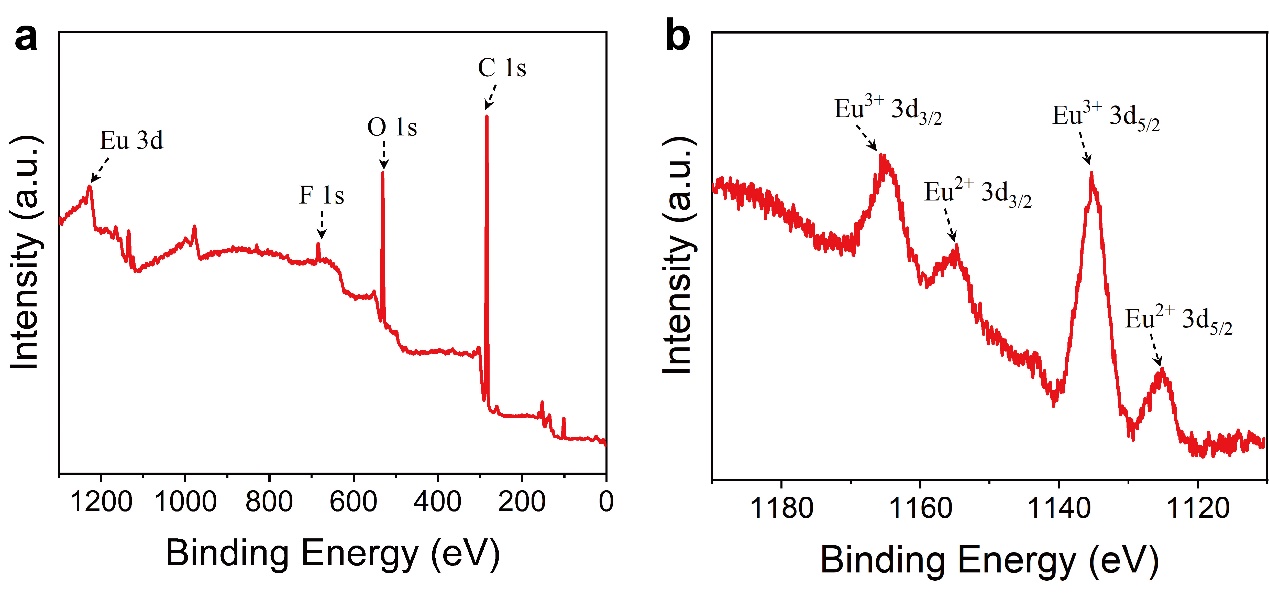


**Figure S8. a** The X-ray photoelectron spectroscopy (XPS) spectrum of LNP:50%Tb@EuSe nanocomposite. **b** The high-resolution XPS spectrum of Eu at 3d position of LNP:50%Tb@EuSe.


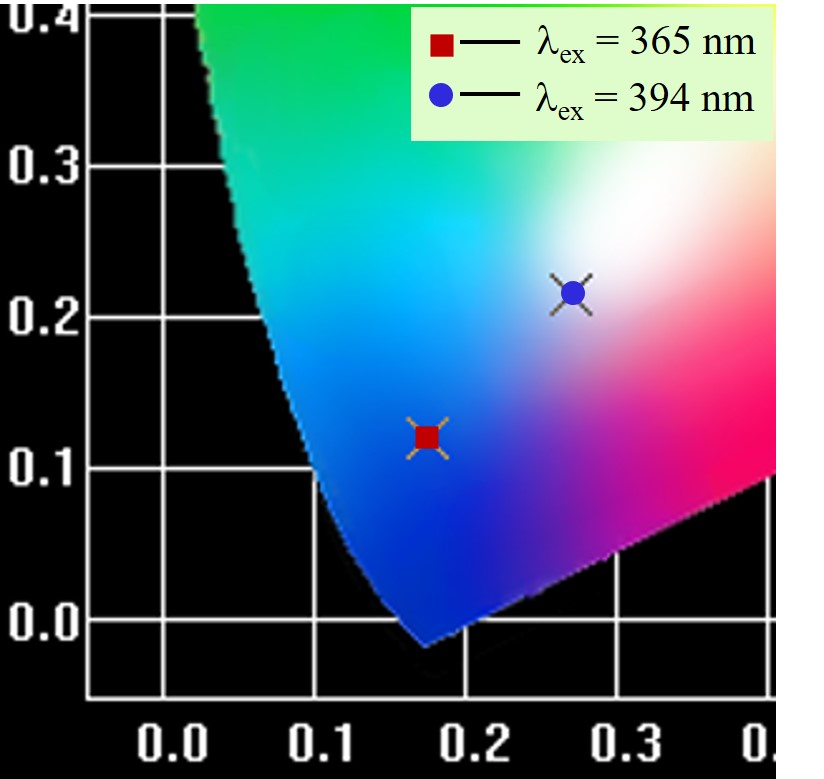


**Figure S9.** The Commission Internationale de l’Eclairage (CIE) chromaticity diagram of LNP:50%Tb@EuSe nanocomposite under 365 nm and 394 nm excitation, respectively.


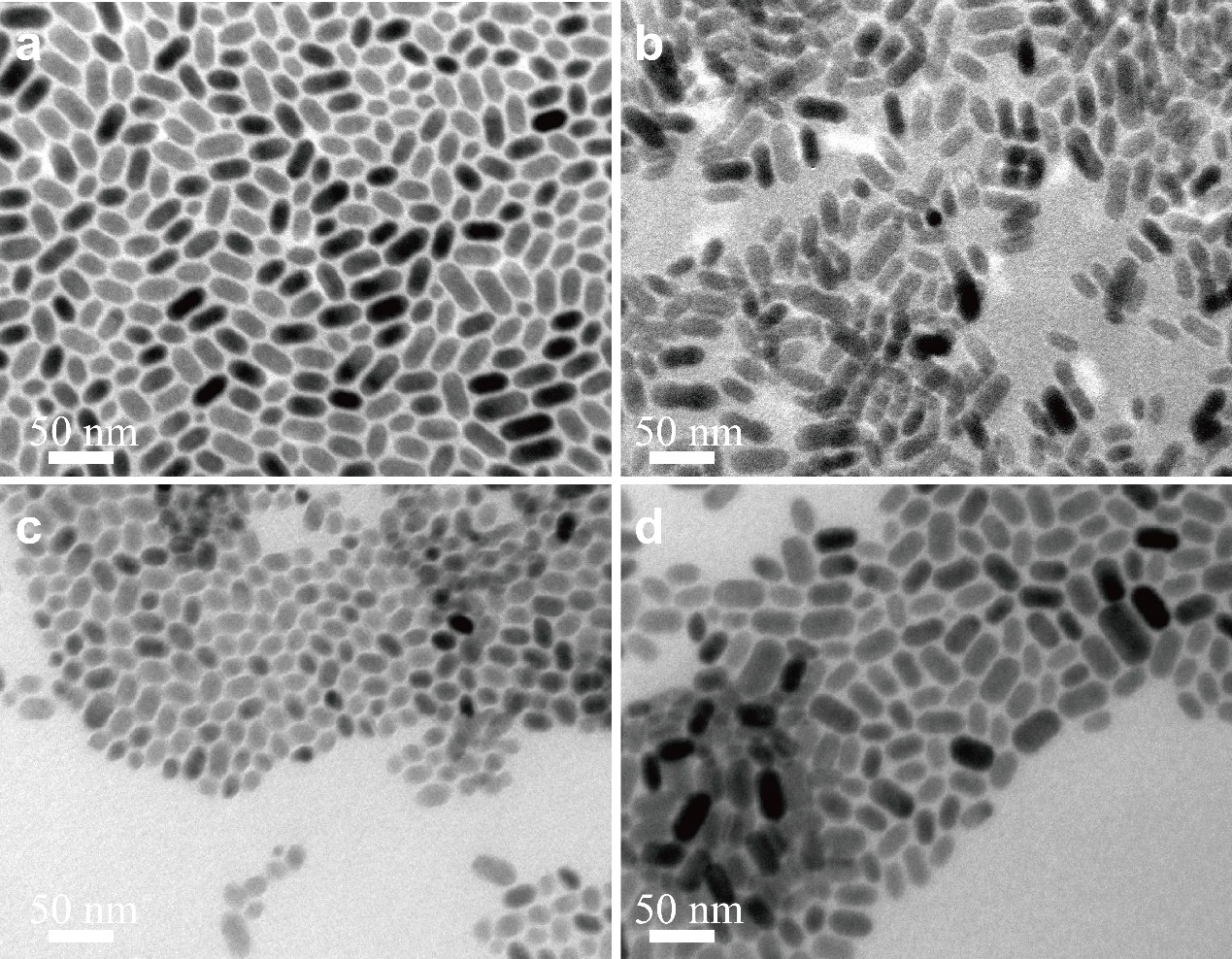


**Figure S10.** The TEM images of NaGdF_4_:Yb,Tm@NaYF_4_:x%Tb@EuSe, denoted as LNP:x%Tb@EuSe, x=10 **(a)**, 30 **(b)**, 70 **(c)**, and 100 **(d)**.


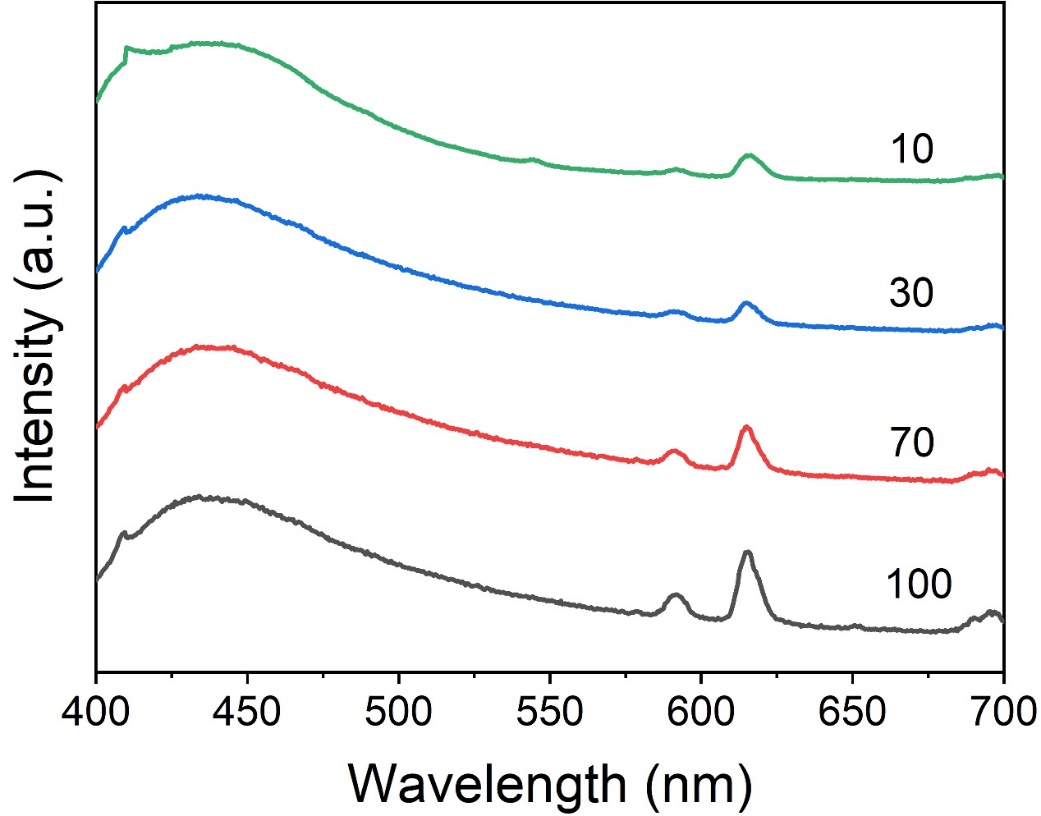


**Figure S11.** The emission spectra of LNP:x%Tb@EuSe under excitation of 365 nm (x = 10, 30, 70, and 100, respectively).

**
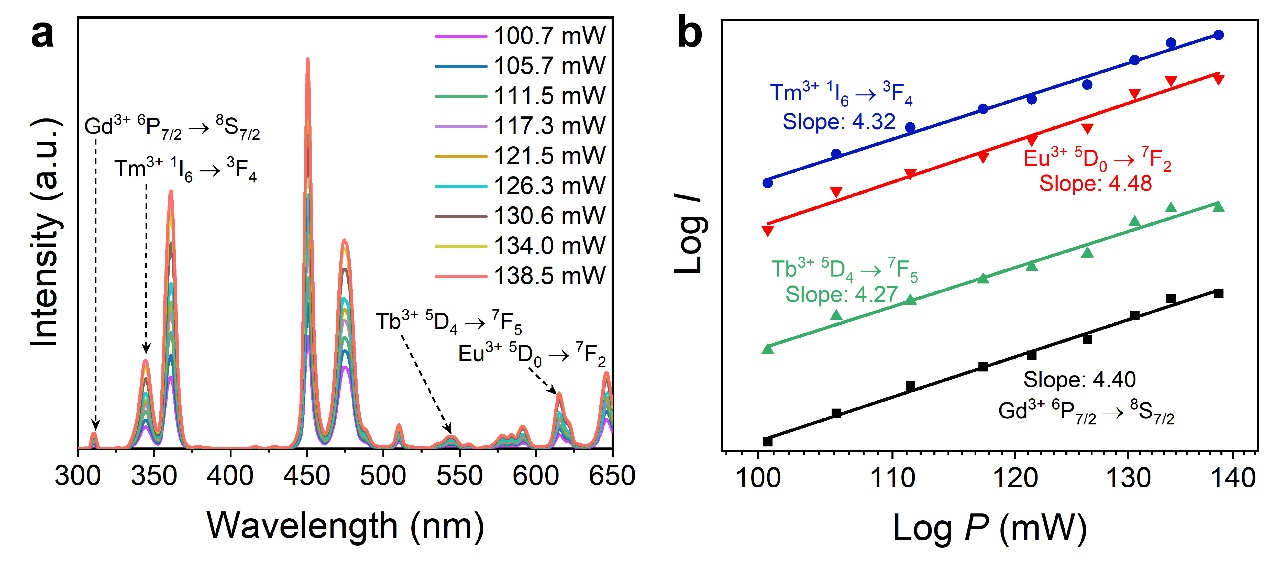
**

**Figure S12.** **a** Upconversion emission profiles of LNP:50%Tb@EuSe nanocomposite under 980 laser excitation with different power. **b** Log–log plots of the upconversion emission intensity against the 980 nm excitation power for ^6^P_7/2_ → ^8^S_7/2_ transition of Gd^3+^, ^1^I_6_ → ^3^F_4_ transition of Tm^3+^, ^5^D_4_ → ^7^F_5_ transition of Tb^3+^, and ^5^D_0_ → ^7^F_2_ transition of Eu^3+^.


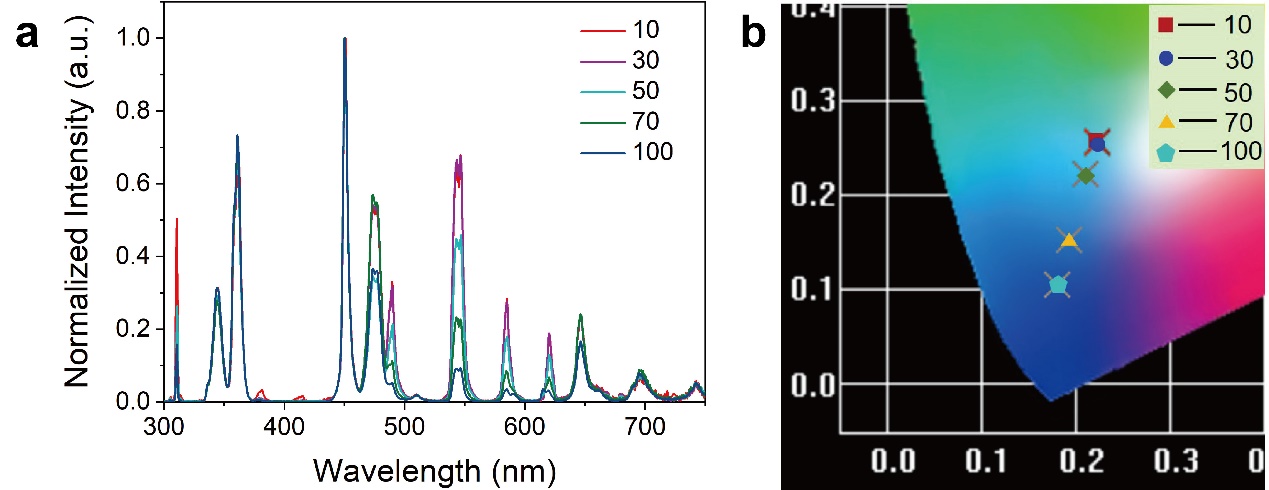


**Figure S13.** The normalized upconversion luminescence spectra **(a)** and the corresponding CIE diagram **(b)** of LNP:x%Tb (x=10, 30, 50, 70, and 100, respectively, λ_ex_ = 980 nm).


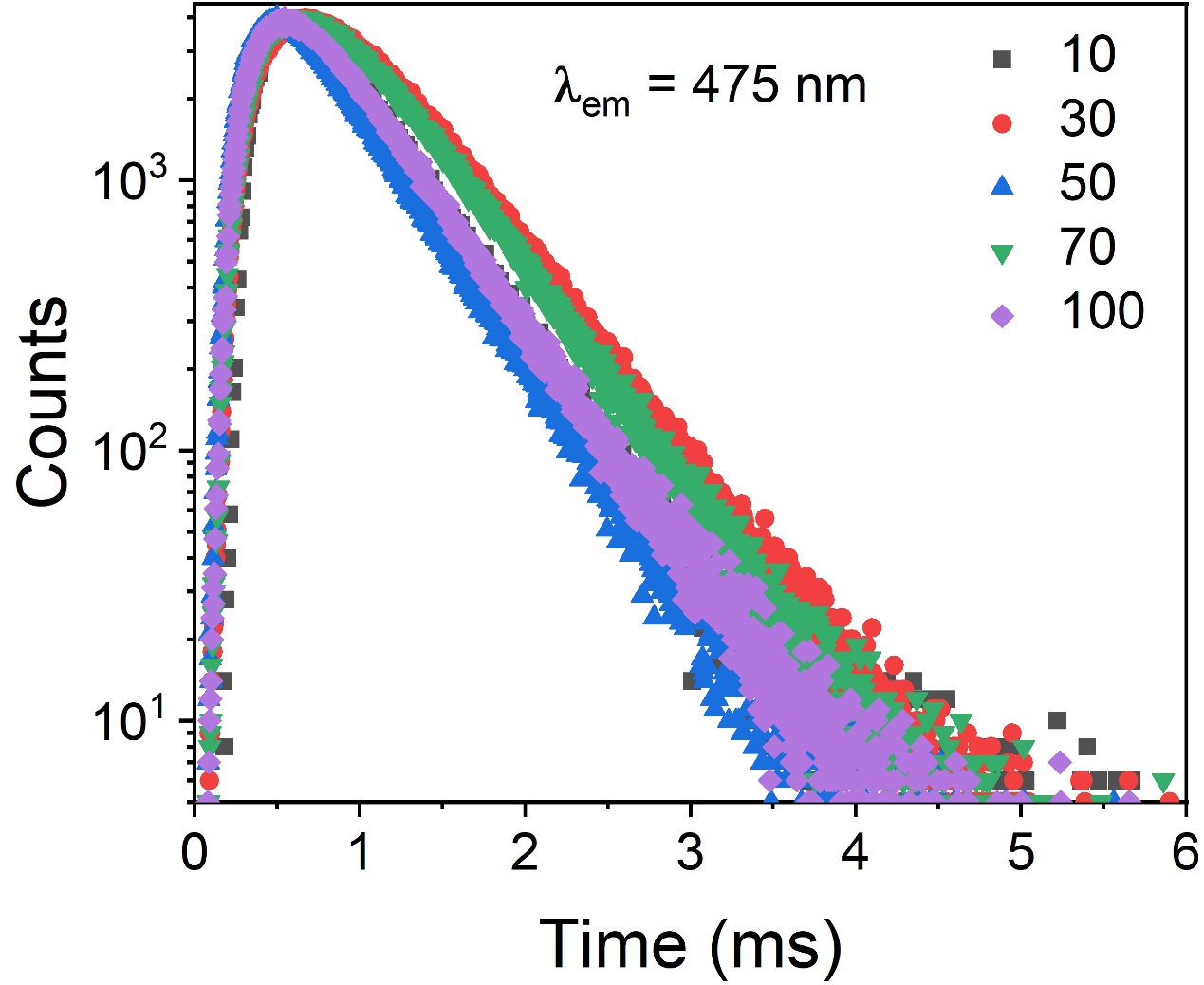


**Figure S14.** Decay curves of Tm^3+^ ion at ^1^G_4_ state (monitored at 475 nm) in LNP:x%Tb@EuSe (x=10, 30, 50, 70, and 100, respectively) under 980 nm pulse excitation.


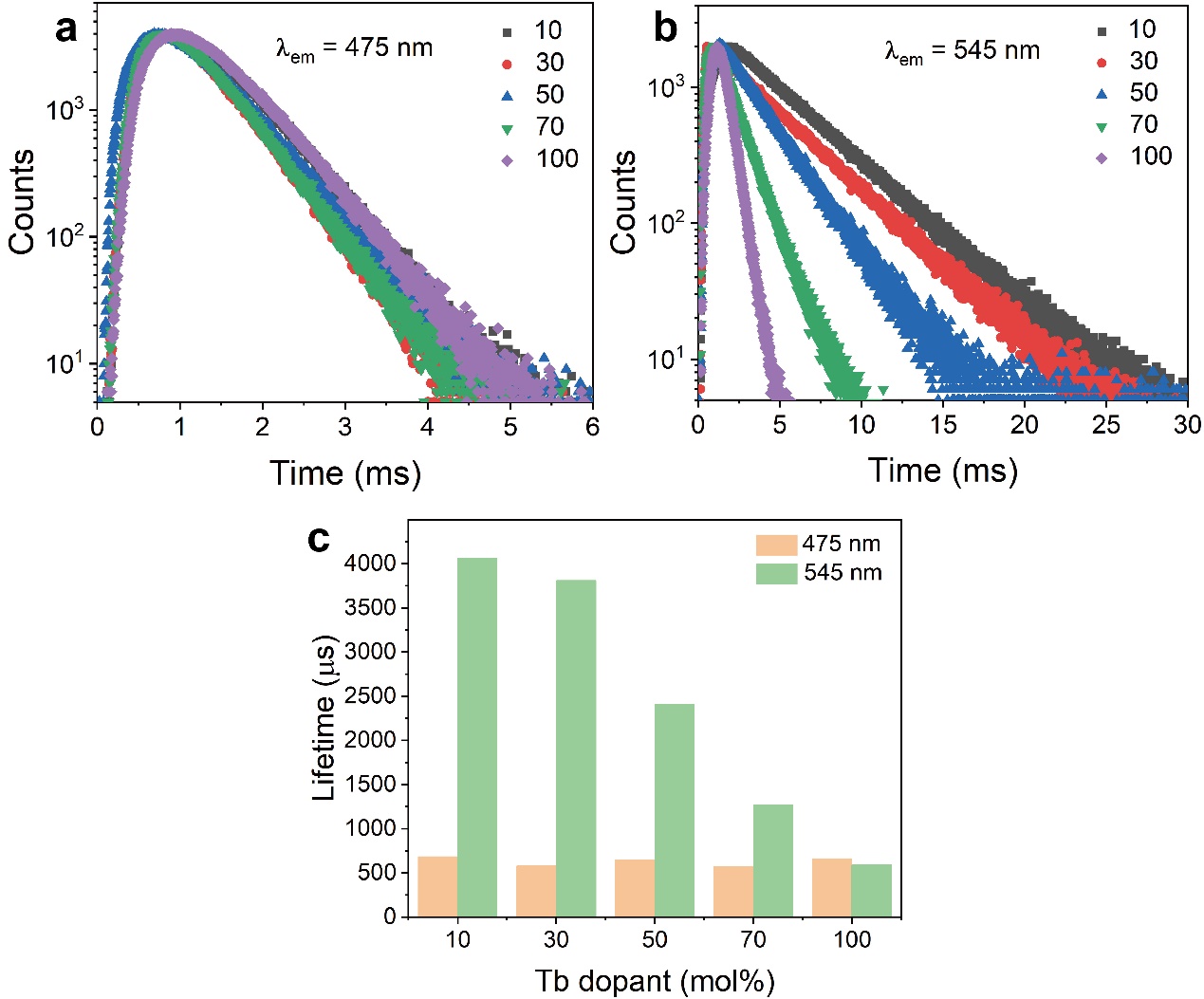


**Figure S15.** Decay curves of Tm^3+^ at ^1^G_4_ state (monitored at 475 nm) **(a)** and Tb^3+^ at ^5^D_4_ state (monitored at 545 nm) **(b)** in LNP:x%Tb (x = 10, 30, 50, 70, and 100, respectively) under pulsed 980 nm excitation, and the histogram of corresponding lifetime values **(c)**.


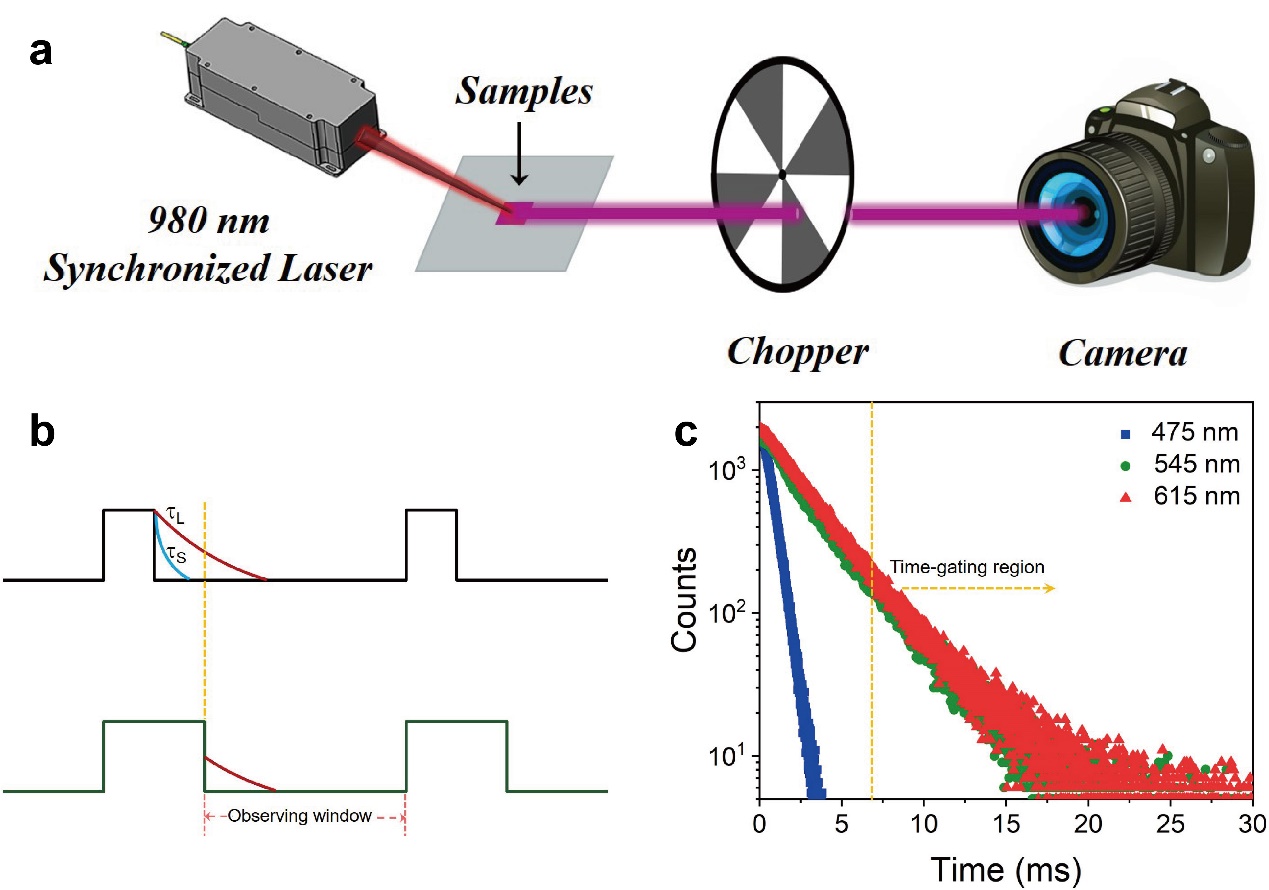
 **Figure S16.** A mechanistic description of lifetime multiplexing using the time-gating technique for fluorescence separation. **a** Schematic diagram of time-gating imaging system. **b** Schematic diagram of filtering short-lived emission (τ_s_) from long-lived emission (τ_L_) by the concatenation of pulse synchronizer and chopper. The delay time is 7 ms and observing time is 10 ms. **c** Decay curves of Tm^3+^ at ^1^G_4_ state (monitored at 475 nm), Tb^3+^ at ^5^D_4_ state (monitored at 545 nm), and Eu^3+^ at ^5^D_0_ state (monitored at 615 nm) in LNP:30%Tb@EuSe under pulsed 980 nm excitation.


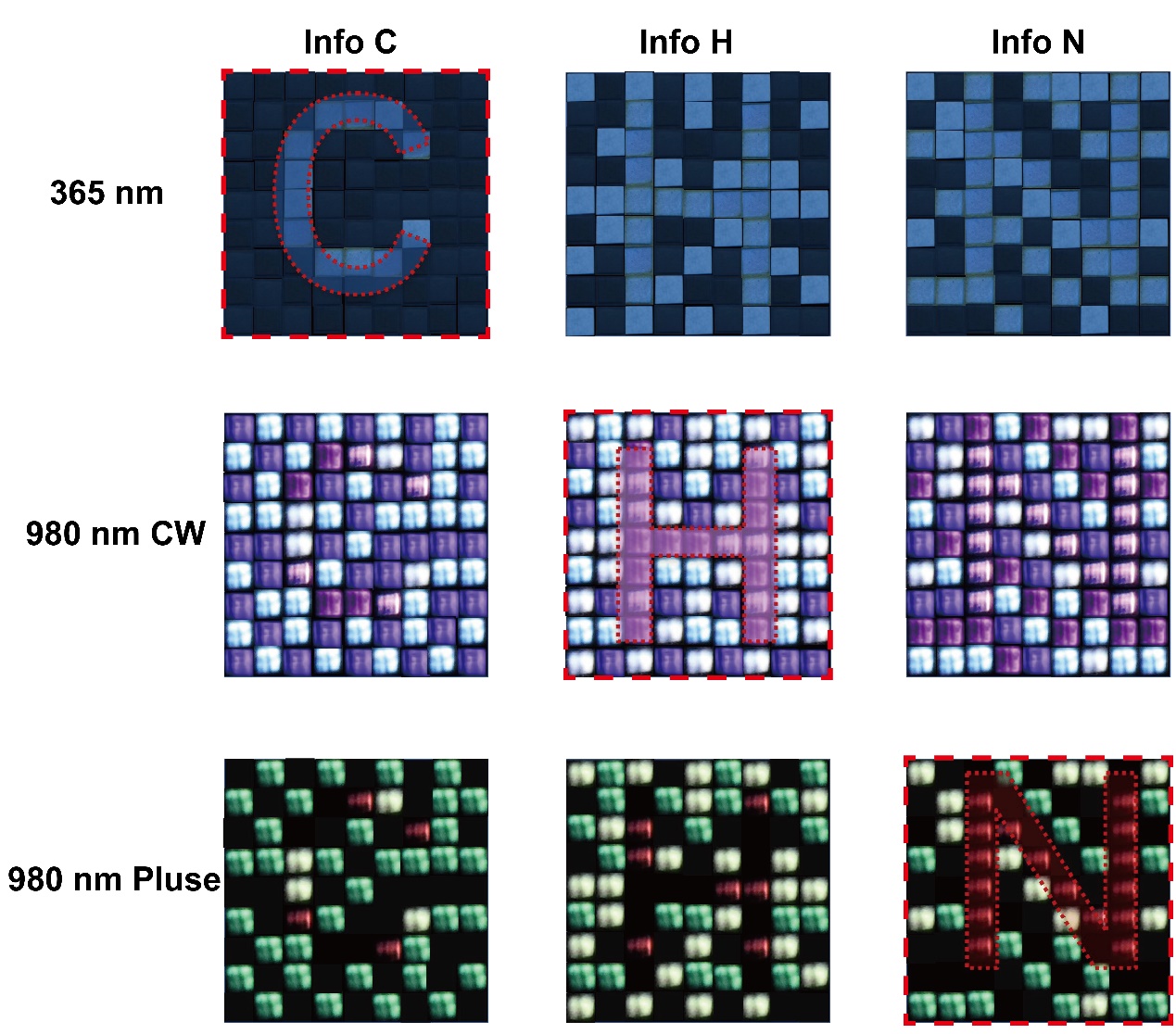


**Figure S17.** The different information was hidden in three different arrays (9 × 9) of optical modules. “C”, “H” and “N” were successfully read under UV (365 nm), 980 nm continuous wave (980 nm CW), and 980 nm pulsed laser (980 nm Pulse) excitation, respectively.

**
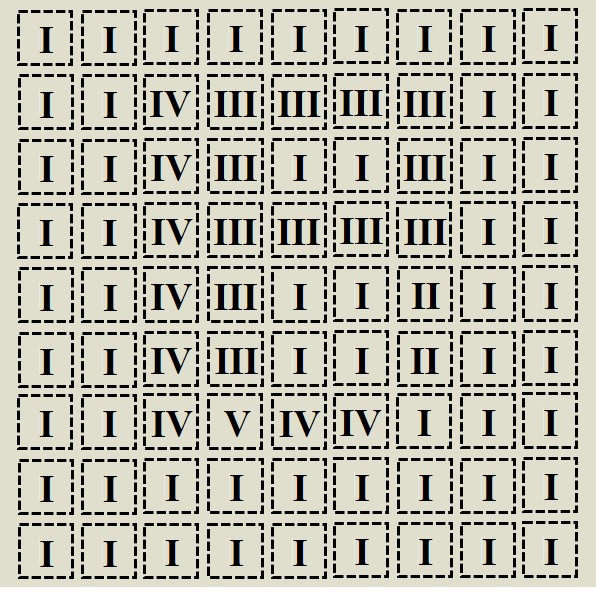
**

**Figure S18.** Schematic diagram of optical modulate array (9 × 9) containing the hidden information of “L”, “A”, and “P” by utilizing nanomaterials I-V (I: LNP:30%Tb@EuSe; II: LNP:50%Tb@EuSe; III: LNP:100%Tb@EuSe; IV: LNP:50%Tb; V: LNP:100%Tb).

**Table S1.** Lifetime values of Tm^3+^ at 475 nm, Tb^3+^ at 545 nm, and Eu^3+^ at 615 nm in LNP:x%Tb@EuSe nanocomposite (x = 10, 30, 50, 70, and 100) under pulsed 980 nm excitation.

| **Tb content (x% mol)** | **τ_475_ (μs)** | **τ_545_ (μs)** | **τ_615_ (μs)** |
| --- | --- | --- | --- |
| 10 | 494.72 | 3288.17 | 2100.62 |
| 30 | 604.43 | 2897.17 | 2986.50 |
| 50 | 484.06 | 848.83 | 2977.18 |
| 70 | 580.58 | 917.92 | 1918.39 |
| 100 | 523.36 | 690.76 | 1527.38 |

**Table S2.** Lifetime values of Tm^3+^ at 475 nm and Tb^3+^ at 545 nm in LNP:x%Tb nanoparticles (x = 10, 30, 50, 70, and 100) under pulsed 980 nm excitation.

| **Tb content (x% mol)** | **τ_475_ (μs)** | **τ_545_ (μs)** |
| --- | --- | --- |
| 10 | 680.33 | 4064.30 |
| 30 | 578.81 | 3809.64 |
| 50 | 649.28 | 2411.39 |
| 70 | 571.65 | 1271.22 |
| 100 | 661.10 | 588.71 |
